# Notes and News

**Published:** 1887-08-13

**Authors:** 


### NOTES AND NEWS.

COLLECTED AND COMMUNICATED.

IN answer to advertisements for designs for the new infectious diseases hospital, to be erected by the joint local boards of Swindon, no less than forty-two sets of plans were sent in by architects residing in all parts of the Kingdom. The committee, after a careful deliberation, have selected the plan of Mr. R. Godfrey, of Silver How House, Kingsheath, Birmingham. Two other plans were reserved in case it should be found that Mr. Godfrey's plan cannot be carried out for the amount stipulated—£1,300, exclusive of site and fencing.

MR. JAMES MUIR, of Abbeybank, presided at the annual meeting of the subscribers to the Arbroath Infirmary, which was held in the town-hall on the 19th ult. The directors' forty-third annual report was submitted, showing that the efficiency of the staff of the house had been fully maintained, that the expenditure during the past year had been less than the previous year, and that the institution continued to be a source of substantial comfort and blessing to many unfortunate sick and wounded people. The hospital was clean, commodious, well furnished, and in good condition; the management of the lady superintendent admirable; the service faithful, diligent, and orderly; the medical attendance completely satisfactory, and the income more than equal to the expenditure. What more does any reasonable person want?

THE twenty-third annual meeting of the governors of the Tewkesbury Royal Hospital was held in the council-chamber on Monday, the Rev. Canon Robeson presiding. There were present also the Mayor (Mr. B. T. Moore), Messrs. C. W. Moore, D. Devereux, F. Moore, W. F. M. March Phillips, W. H. Maund, C. Tysoe, E. W. Edgwick, W. North, J. C. Coleman, R. Chandler (secretary), and Captain Freeman. The report of the institution, which was read, showed that the usual good work had been done, and that the hospital had never been overshadowed by pecuniary difficulties.

THE Bute Hospital, Luton, being rather deficient in funds, a festival is suggested for the purpose of raising the wind. The secretary is of opinion that a harvest-home celebration with a procession, accompanied by prayer and dancing, may prove attractive. The secretary is probably a man of rather versatile tastes, but we question whether the combination of harvest home, procession, praying and dancing, will contribute very much either to the funds of the hospital or to the esteem in which its officials are held in the neighbourhood.

THE annual meeting of the governors and subscribers of the Birmingham and Midland Eye Hospital was held on Thursday in the board-room, under the presidency of the Mayor, Sir Thomas Martineau. The report of the general committee for the year ending March 31st, which was read by the Rev. B. J. Bateman, stated that notwithstanding the continued trade depression, and the notable falling off of subscriptions to all London as well as provincial medical charities, their own list exhibited a steadily increasing progress. The managers of the hospital are to be congratulated on this highly satisfactory condition of things, and if they have any royal road to success, or easy Primer of Principles of Management, they might publish them for the advantage of other institutions.

MR. HUCKS GIBBS presided at Guy's Hospital over a meeting of subscribers to the Special Appeal Fund. Among those present were Sir Henry Peek, Mr. Bonsor, M.P., Mr. Arthur Cohen, Colonel Curtis Hayward, Mr. Golding Bird, Mr. David Powell, and Mr. Arthur Mills. The chairman stated that the fund amounted to £75,000. The meeting, it appeared, had been called in consequence of the subscribers having been informed that their funds would be employed in keeping open the hospital, and maintaining a number of beds on the present basis. The Charity Commissioners, however, had informed the governors that a considerable sum having been collected, an over-draft at the bankers' must be paid off, and that sanction would not be given to the borrowing of more money. The actuary was consulted, and it was found advantageous to pay off the debt at once. This reduced the £75,000 to something under £60,003. The interest on that sum, however, was not sufficient to maintain 500 beds, but it was hoped that it would be possible to save the principal, and that there would be increased subscribers in future, so that, interest and subscriptions combined, the work of the hospital might be satisfactorily carried on. This plan met with general approval.

The following vacancies are announced this week, the amount of the salary being placed in each case after the name of the institution :—

**Resident Medical Officers.**

British Columbia. Medical Missionary.—£150.
Durness, Sutherlandshire Parish Medical Officer.—£150.
Female Lock Hospital, Harrow Road, W. Assistant House Surgeon.—No salary.
Leeds Infirmary. One. Qualified.—£100.
Mediterranean Winter Station. Doctor Wanted. Apply to R. Bradshaw & Co., Imperial Buildings, Ludgate Circus.
Pennygown and Torosay. Medical Officer.—£100.
Salford Union. Assistant Medical Officer. Doubly-qualified. —£140.
Sunderland Infirmary. House Physician. Doubly-qualified. —£80.
Ventnor Hospital for Consumption. Assistant.
Warrington Infirmary. Senior and Junior Surgeon-Apothecaries.—£100 and £80 respectively.

**Non-resident Medical Officers.**

Bristol Royal Infirmary. Obstetric Physician.
City of London Hospital for Diseases of the Chest, Victoria Park, E. Assistant Physician.
Newcastle-on-Tyne. Visiting Medical Assistant. Doubly-qualified.—£120.
Lampeter Union. Medical Officer of Health.—£60.
West End Hospital for Paralysis, 73, Welbeck Street. Physician.

**Matron.**

Manchester, Monsall Fever Hospital. Lady Superintendent. —£80.

**Trained Nurses.**

Birmingham Children's Hospital. Assistant Nurse.
Bradford Infirmary. Assistant.—£16.
Bridgewater Infirmary. Charge Nurse.—£25.
Brighton Infectious Hospital.—£23.
Cambridge, 13, Fitzwilliam Street. Private Nursing.—£25.
Coventry. Charge of Paralysed Gentleman. S. Berry, Radford Street.—£30.
Denbighshire Infirmary.—£18.
Hertford General Infirmary. One.—£2 2s. per month.
Home for Nurses, 37, Monkgate, York. Private work.
Kingston Home, Clare House, Hull. Several.
London, 27, Margaret Street, W. Several.
Nottingham. Surgical, Medical, and Monthly Private Nurses. Lady Superintendent, 1, Regent Street, Nottingham.
Victoria Hospital, Burnley, Lancashire. Head Nurse, Women and Children's Ward.—£26.
Wimborne. Parish Nurse. Not over forty years of age.
West Bromwich. One.—£22.
Workington Infirmary.—£18.
Worthing Infirmary.—£20.

---

The Beach Rock Convalescent Home of the London Samaritan Society at Sandgate has just been enlarged. The step was rendered necessary by the increased number of applications for admission to the Home. The Vicar of Sandgate, the Rev. H. Tindal, with Mr. John James Jones, the Director of the Society, and other gentlemen inspected the rooms of the new buildings, and the vicar conducted a short service amongst the patients at Beach Rock. The assembled inmates, to the number of about eighty, expressed their thanks to the vicar for his kindness, and to the Earl of Chichester for his frequent presents of fruit, vegetables, honey, etc., to the institution.

---

The Lowestoft Convalescent Home has secured for itself warm friends in its own locality. A local contemporary says :—"That the Home has proved a great success goes without saying; and no wonder, when we remember those at the head of it, and the liberal treatment the inmates receive. The names of Birkbeck, Lucas, and others have but to be mentioned to inspire the utmost confidence, and we cannot wonder, therefore, at the popularity the institution has attained ; and the continual applications for admission which are being made prove this fact in a remarkable manner."

In the country the general public seem to take a much more lively interest in hospitals than in some of the largest towns. On Saturday, the 23rd ult., the annual Deighton and Sheepridge demonstration for the benefit of the Huddersfield Infirmary took place. A procession was formed at Deighton, consisting of Oddfellows, Foresters, and Buffaloes. The procession marched to Huddersfield and thence to the Deighton cricket-ground, and on the way men on foot and horseback made a collection for the infirmary. Unfortunately a drenching rain, which continued more or less till dusk, marred the proceedings and diminished the numbers. There was a horse-show, an assault-at-arms, a cricket match, and tug-of-war, with a variety of other sports. More than 2,000 tickets of admission were sold before Saturday. Last year nearly £50 was realised by the demonstration, and handed over to the funds of the infirmary.

---

Wellingborough, which has long suffered from the want of a cottage hospital, is rousing itself with a view to the foundation of such an institution. The large numbers of comparatively poor and ill-paid shoe-operatives at Wellingborough have often to endure great privations for want of skilled medical attendance, in spite of the several medical clubs which have for some time flourished there. The kind of club which prevails at Wellingborough is of the most unsatisfactory character, due to the action of local medical men, who have not sufficiently restricted the area of the operations of such clubs. The result has been that medical practice in the town has suffered both in emolument, efficiency, and status. It is much to be hoped that the establishment of a sufficiently large and well-managed cottage hospital will put an end to a very discreditable state of things.

---

The *Liverpool Courier*, commenting on certain charges brought by Dr. H. Pitts of Tuebrook against the administration of the Liverpool Royal Infirmary, says :— "As Dr. Pitts is himself an *alumnus* of that institution, it cannot be supposed that he is animated by any desire to depreciate its reputation, rather we should imagine him to be anxious if possible to vindicate and rehabilitate it. While therefore we hesitate to jump to a conclusion in the matter until we have heard what is to be said on the other side, we cannot refrain from expressing our opinion that the charges of incivility and incapability which he brings against certain officers of the institution demand careful investigation at the hands of the authorities, the more so that as regards incivility at all events they are entirely in accordance with reports that have reached us from other quarters. Medical students have never been particularly celebrated for good breeding, and though there has doubtless been an enormous improvement since the days of Mr. Bob Sawyer and Mr. Ben Allen, yet it will still follow that if young men, a little conceited probably at their success, are placed in honorary positions of considerable responsibility and authority, they will develop no small amount of the perfunctory jack-in-office spirit, of which Dr Pitts complains." It is not pleasant to read of such strange behaviour as is charged by Dr. Pitts upon some of the officials at the Liverpool Infirmary, and it may be trusted that the responsible managers will at once institute a thorough inquiry into the circumstances.

A SPECIAL Hospital for Diseases of the Throat and Ear is proposed to be erected at Nottingham. A meeting was recently held, convened by circular, at which Mr. Alderman Turney, J.P., the Mayor, presided; and there were present the Rev. G. Edgcumbe, the Rev. J. F. Wilkenson, the Rev. Robert Cowen, the Rev. P. Smith, the Town Clerk (Mr. S. G. Johnson); Messrs. G. Barrows, W. H. Lee, H. Hill Adcock, and Drs. Withers, Macdonald, Cattle, Stafford, Stewart, and Mutch. The Rev. G. Edgcumbe moved that a committee be appointed to take the necessary steps for the establishment of the proposed hospital. The town-clerk seconded, and the resolution was agreed to. Would it not be better to add an efficient sub-department to the existing hospital at Nottingham, rather than to erect a new hospital, with new officials of all kinds, and great pecuniary responsibilities? It is a pity to found special hospitals in any town or district where it is possible to affiliate a sub-department to an existing institution.

---

THE Earl of Clarendon presided at the Annual Meeting of the subscribers to the Watford District Cottage Hospital on Tuesday. There were present Mr. J. F. Watkins, Mr. J. W. Robins, Mr. Holland-Hibbert, Mr. G. Wailes, Dr. Brett, Mr. G. Rooper, Mr. H. M. Turner, Mr. C. E. Keyser, Mr. E. J. Slinn, Mr. R. Savill, Mr. Marnham, Mrs. Watkins, Mrs. Robins, Mrs. Rivaz, Mrs. Morten Turner, Miss Savill, etc., etc. The hospital has been in operation one year, and the committee consider that the history of the year's work is in every respect satisfactory. It is a circumstance worthy of note that the medicines required have been supplied by a local chemist, and this arrangement has been found to work well. The income of the hospital has been fairly adequate, and the treasurer has a small balance in hand. The endowment fund, which amounted to £2,436 at the beginning of the year, has been increased by £200 by the liberality of Miss Hudson, and by various Jubilee donations, amounting altogether to £433 4*s.* 2*d.* This is a very gratifying record for a young institution.

---

ON Friday the Annual Court of Governors of the Sunderland and Bishopwearmouth Infirmary was held in the public room of the hospital, under the presidency of Mr. James Laing, J.P. There were also present the Venerable Archdeacon Long, the Mayor, E. Richardson, Esq., the ex-Mayor, Alderman Preston, Mr. I. Johnston, Mr. W. M. Roche, Mr. A. A. Webster, Mr. J. C. Pattison, Rev. J. J. Browne, Dr. Morgan, Dr. Whitehouse, and others. The chairman congratulated the meeting on the fact that during the year there had been an amalgamation of the two institutions, the Infirmary and the Children's Hospital, and also on the success of the efforts of Alderman Preston on behalf of the new Hartley wing, the building of which, he hoped, would be commenced forthwith. He considered it an occasion for special satisfaction that several collieries had taken up the cause of the hospital in good earnest, and that the working men of the district had surpassed all their former efforts on its behalf. The report showed the hospital to be in a prosperous and efficient condition.

THE amount subscribed during the past year by the working men of Sunderland towards the funds of the Infirmary was £2,400. The chairman at the Annual Meeting expressed the opinion that this munificence on the part of the operatives was the more remarkable, because the collections at the churches and chapels had shown a considerable falling off. It is complained, and apparently with justice, that the wealthier inhabitants of Sunderland are inclined to be stingy towards their hospital. Perhaps the collector calls upon them immediately before dinner. Let him try a call when the "walnuts and the wine" have been sufficiently discussed.

---

THERE must be a majority of Irishmen on the committee of the Princess Alice Memorial Hospital at Eastbourne. True to their Hibernian instincts, they have, no doubt with good intentions, turned the only available outdoor recreation space of the hospital into a potato ground! We ourselves confess to a liking for potatoes, especially new potatoes newly dug out of our own garden; but it seems to us the most mistaken economy which any body of men could practise, to turn one of the best means of health which a hospital possesses to the poor use of potato-growing. Every hospital should have a large open space at its disposal, for outdoor exercise for the patients. The increased rapidity of recovery which would result would counterbalance a hundred times over any little extra expenditure at the greengrocer's. The Eastbourne committee should purchase a few elementary books on health.

---

THEIR Royal Highnesses the Duke and Duchess of Connaught, accompanied by Princess Margaret, visited Westminster Hospital, to open a children's ward in connection with that institution. Archdeacon Farrar said the new ward was used for many years as the chapel of the hospital, but as a fresh chapel was built last year the ward had become available for the reception of patients, and the committee had decided to use it as a children's ward. The inhabitants of Westminster, of their own accord, have determined to raise a fund for the endowment of children's cots as a memorial of her Majesty's Jubilee. Four out of the twelve cots have been already endowed. The Princess Margaret shook hands and kissed several of the occupants of the cots. One of the little girls, raising her doll in her hands, said, "May it please your Royal Highness, will you please accept this cot and doll from the children of the Westminster Hospital?" At the close of the proceedings their Royal Highnesses were cordially thanked for their presence.

---

THE Rev. A. Isham presided at the twenty-first Annual Meeting of the Reigate and Redhill Cottage Hospital on Thursday evening, in place of Mr. St. Barbe Solden, who telegraphed inability to be present. There were also present Rev. G. J. Adeney, Mr. A. C. Sterry, Mr. William Allingham, Mr. A. Murray, and Mr. J. J. Rapley, secretary. The income of the hospital for the year was £1,206 18*s.* 1*d.*, which exceeded the expenditure by £24 17*s.* 1*d.* Dr. Walters, Dr. Stone, Mr. W. A. Berridge, and Mr. Ewon were re-elected on the medical staff for the ensuing year, and Mr. Alderman Yerworth was reappointed auditor.

